# A new species, a new combination, and a new record of *Crossotarsus* Chapuis, 1865 (Coleoptera, Curculionidae, Platypodinae) from China

**DOI:** 10.3897/zookeys.1028.61018

**Published:** 2021-04-06

**Authors:** Shengchang Lai, Ling Zhang, You Li, Jianguo Wang

**Affiliations:** 1 College of Forestry, Nanjing Forestry University, Nanjing, Jiangsu 210037, China Nanjing Forestry University Nanjing China; 2 College of Agricultural Sciences, Jiangxi Agricultural University, Nanchang, Jiangxi 330045, China Jiangxi Agricultural University Nanchang China; 3 School of Forest Resources and Conservation, University of Florida, Gainesville, FL, 32611, USA University of Florida Gainesville United States of America

**Keywords:** Ambrosia beetle, Fujian, Jiangxi, molecular phylogeny, pinhole borer, taxonomy

## Abstract

This study describes a new species, *Crossotarsus beaveri* Lai & Wang, **sp. nov.**, designates a new combination, *C. brevis* (Browne, 1975, **comb. nov.** from *Platypus* Herbst, 1793), and notes a new record, *C. emorsus* Beeson, 1937, from China. Genetic data from four genes indicate that the new species and *C. brevis* form a clade clustered with other *Crossotarsus* species. Molecular phylogeny and morphological characters support their taxonomic placement.

## Introduction

The genus *Crossotarsus*Chapuis 1865 was erected for 29 species of pinhole borers. *Crossotarsus wallacei* (Thomson, 1857) was designated as the type species of the genus (Hopkins 1914). Wood (1993) revised the genera of Platypodidae and placed *Crossotarsus* in the subfamily Platypodinae, tribe Platypodini. *Crossotarsus* is distinguished from other Platypodine genera primarily by the following combination of characters (Browne 1961; Wood 1993; Beaver and Sanguansub 2015): 1. Labial palps two-segmented, with basal segments fused in the midline; 2. Sexually dimorphic protibiae, the outer face of the protibia transversely carinate in the male and finely granulate in the female; 3. Pronotum without specialized mycangial pores in either sex; 4. Femoral grooves angulate at the anterior extremity and gently rounded behind; 5. Metacoxa strongly projecting with a deep vertical posterior face. But Wood’s (1993) generalisation that the female pronotum of *Crossotarsus* species has numerous mycangial pores is incorrect (Beaver 2004).

The catalog of Wood and Bright (1992) includes 118 species of *Crossotarsus*. As a result of taxonomic changes since that time, 116 species are currently recognised. Most species of *Crossotarsus* occur in the Oriental region, extending from India across Southeast Asia and Indonesia to Australia and the Pacific islands, and northward to Taiwan and Japan (Wood 1993). *Crossotarsus externedentatus* (Fairmaire 1849) is also widespread in the Afrotropical forests.

The Platypodinae have been almost entirely neglected in China. Only a few papers include original records of *Crossotarsus* from the country. Yin and Huang (1987) recorded three species: *C. coniferae* Stebbing, 1906, *C. squamulatus* Chapuis, 1865, and *C. wallacei* from Yunnan. Yin et al. (2002) added two species: *C. externedentatus* and *C. terminatus* Chapuis, 1865 from Hainan island, and Zhang et al. (2008) cited 13 species from China. After taxonomic changes (Beaver 2004; 2005; 2016; Bright 2014), the following 13 species are currently known from China: *C. coniferae* (Yunnan, Sichuan, Xizang); *C. emancipatus* Murayama, 1934 (Taiwan); *C. externedentatus* (Hainan, Taiwan); *C. flavomaculatus* Strohmeyer, 1912 (Taiwan); *C. formosanus* Strohmeyer, 1912 (Taiwan); *C. niponicus* Blandford, 1894 (Taiwan); *C. piceus* Chapuis, 1865 (Taiwan); *C. saltatorinus* (Schedl, 1954) (Fujian); *C. sauteri* (Strohmeyer, 1913) (Taiwan); *C. simplex* Murayama, 1925 (Taiwan); *C. squamulatus* (Yunnan); *C. terminatus* (Hainan, Yunnan, Xizang); *C. wallacei* (Hainan, Taiwan).

In this study, we describe a new species of *Crossotarsus* from China, give a new distribution record, transfer a previously described species to the genus, and provide molecular data of Chinese species for molecular phylogenetic analyses.

## Materials and methods

### Abbreviations used for collections

**BMNH**The Natural History Museum, London, United Kingdom.

**JXAU**College of Agricultural Sciences, Jiangxi Agricultural University, Nanchang, China.

**KIZCAS**Kunming Institute of Zoology, Chinese Academy of Sciences, Kunming, China.

**NIAES**National Institute of Agro-Environmental Sciences (ITLJ), Tsukuba, Ibaraki, Japan.

**NMNS**National Museum of Natural Science, Taichung, Taiwan.

**NZMC** National Zoological Museum of China, Institute of Zoology, Chinese Academy of Science, Beijing, China.

**RAB** Private collection of Roger A. Beaver, Chiang Mai, Thailand.

**RIFID** Research Institute of Forest Insect Diversity, Namyangju, South Korea.

**SYU** Museum of Biology, Sun Yat-sen University, Guangzhou, China.

**USNM**National Museum of Natural History, Washington D.C., USA.

**ZIN** Zoological Institute. Russian Academy of Sciences, St. Petersburg, Russia.

Adults of the new species were collected by log dissection. The samples were immediately preserved in tubes containing 99.9% ethyl alcohol, which were stored at −20 °C for DNA extraction and examination. Specimens were examined using an Olympus SZX160 stereoscopic zoom microscope. Photographs were taken with a KEYENCE VHX-6000 Digital Microscope System. All photos were further adjusted and assembled with Adobe Photoshop CS6. Body length was measured between the anterior margin of the pronotum and the elytral apex (head not included).

Genomic DNA was extracted from the adult head. The total genomic DNA was extracted from each individual using the Ezup Column Animal Genomic DNA Purification Kit (Sangon Biotech Co. Ltd). Amplification of four gene fragments (COI, EF-1α, CAD, 28S) was made by PCR, using primers (Table 1) and cycling conditions previously described (Jordal et al. 2011). The PCR products were sent to Sangon Biotech Co. Ltd (Shanghai, China) for sequencing, and the sequences were analyzed using the software DNAstar. Additional information on *Crossotarsus* material was collected by the authors in China or downloaded from NCBI (The National Center for Biotechnology Information) (Table 2). Concatenated DNA sequence data from Jordal (2013) were analysed in MrBayes v. 3.2.6 (Ronquist et al. 2012). Partitions and models were estimated by PartitionFinder 2 (Lanfear et al. 2017) and ModelFinder (Kalyaanamoorthy et al. 2017) respectively in PhyloSuite (Zhang et al. 2020), GTR+G+I were selected for each partition. 10 million generations were run, with 25% of the generations as burn-in. PSRF close to 1.0 and standard deviation of split frequencies below 0.01 were accepted.

**Table 1.** Gene fragments targeted for PCR and the primers used. Sequencing primers were identical to those used in PCR.

Gene	Primer name	Annealing	Primer sequence	Reference
COI	S1718	46	5'-GGAGGATTTGGAAATTGATTAGTTCC-3'	Jordal et al. 2011
	A2411		5'-GCTAATCATCTAAAAACTTTAATTCCWGTWG-3'	
28S	S3690	55	5'-GAGAGTTMAASAGTACGTGAAAC-3'	Jordal et al. 2011
	A4394		5'-TCGGAAGGAACCAGCTACTA-3'	
EF-1α	S149	52	5'-ATCGAGAAGTTCGAGAAGGAGGCYCARGAAATGGG-3'	Jordal et al. 2011
	A1043		5'-GTATATCCATTGGAAATTTGACCNGGRTGRTT-3'	
CAD	CAD for4	50	5'-TGGAARGARGTBGARTACGARGTGGTYCG-3'	Jordal et al. 2011
	CAD rev1mod		5'-GCCATYRCYTCBCCYACRCTYTTCAT-3'	

**Table 2.** Material used for phylogenetic analyses, including their GenBank accession numbers.

No.	Taxon	Country	CAD	COI	EF-1α	28S	Reference
1	*C. beaveri* sp. nov.	China: Jiangxi	LC616080	LC613149	–	LC613157	This study
2	*C. brevis* (Browne, 1975)	China: Yunnan	LC616086	LC613154	LC616520	LC613163	This study
3	*C. chalcographus* Schedl, 1972	Papua New Guinea	KR261163	KR261313	–	–	Jordal 2015
4	*C. emorsus* Beeson, 1937	China: Yunnan	LC616087	LC613155	–	LC613164	This study
5	*C. externedentatus* (Fairmaire, 1849)	China: Yunnan	LC616083	LC613152	LC616518	LC613160	This study
6	*C. externedentatus* (Fairmaire, 1849)	Tanzania	KR261162	KR261312	–	KR261216	Jordal 2015
7	*C. externedentatus* (Fairmaire, 1849)	Madagascar	KR261166	KR261316	KR261275	KR261218	Jordal 2015
8	*C. fractus* Sampson, 1912	Papua New Guinea	KR261165	KR261315	KR261274	–	Jordal 2015
9	*C. minusculus* Chapuis, 1865	Papua New Guinea	HQ883809	HQ883669	HQ883739	HQ883579	Jordal 2015
10	*C. niponicus* Blandford, 1894	China: Sichuan	–	LC613156	–	LC613165	This study
11	*C. nitescens* Schedl, 1979	Australia	KR261161	KR261311	KR261272	–	Jordal 2015
12	*C. sauteri* (Strohmeyer, 1913)	China: Jiangxi	LC616081	LC613150	LC616516	LC613158	This study
13	*C. squamulatus* Chapuis, 1865	China: Yunnan	LC616084	LC613153	–	LC613161	This study
14	*C. terminatus* Chapuis, 1865	China: Jiangxi	LC616082	LC613151	LC616517	LC613159	This study
15	*C. wallacei* (Thomson, 1857)	China: Yunnan	LC616085	–	LC616519	LC613162	This study
16	*P. contaminatus* (Blandford, 1894)	China: Jiangxi	LC387560	LC383433	LC387562	LC386151	Lai et al. 2019

## Results

### Crossotarsus beaveri

Taxon classificationAnimaliaColeopteraCurculionidae

Lai & Wang sp. nov.

67FDD7E9-B31C-5E08-BED2-40FAABAFC514

http://zoobank.org/B8D65F2C-90C7-4B5B-84D0-AA714D42A565

Figures 1 , 2

#### Type material.

***Holotype.*** Male, China: Jiangxi Province, Ganzhou City, Longnan County, Jiulianshan national nature reserve of Jiangxi, Hualu Village, 24°37'19"N, 114°29'57"E, 2.VII.2020, log dissection, host *Paulownia fortunei*, Shengchang Lai leg. (deposited in NZMC IOZ(E)225775).

***Allotype.*** Female, same data as holotype (deposited in NZMC IOZ(E)225776).

***Paratypes.*** 6 males, 6 females, same data as holotype, but host *Phoebe zhennan* and *Liquidambar formosana* (5 males, 5 females JXAU; 1 male, 1 female NZMC); 11 male, 6 females, as holotype except: Xunwu County, Xiangshan Town, Congkeng Village, 24°54'20"N, 115°52'44"E, ca 650m, 15.IX.2017, log dissection, host *Castanopsis fargesii* and *Vernicia montana*, Shengchang Lai leg. (10 males, 5 females JXAU; 1 male, 1 female RAB); 6 males, 6 females, as holotype except: Xunwu County, Liuche Town, Luanluozhang, 24°40'41"N, 115°44'9"E, ca 640 m, 22.VIII.2017, log dissection, host *Castanopsis carlesii*, Shengchang Lai leg. (5 males, 5 females JXAU; 1 male, 1 female RAB); 38 males, 38 females, China: Fujian Province, Zhangzhou City, Yunxiao County, Xiahe Town, Qigaoqi Village, 24°1'31"N, 117°10'36"E, 8.VII.2019, log dissection, host *C. carlesii*, Ling Zhang leg. (2 males, 2 females BMNH; 2 males, 2 females KIZCAS [KIZ0121459–0121462]; 2 males, 2 females NIAES; 2 males, 2 females NMNS; 2 males, 2 females RAB; 2 males, 2 females RIFID; 2 males, 2 females SYU; 2 males, 2 females USNM; 2 males, 2 females ZIN; 20 males, 20 females JXAU).

**Figure 1.**
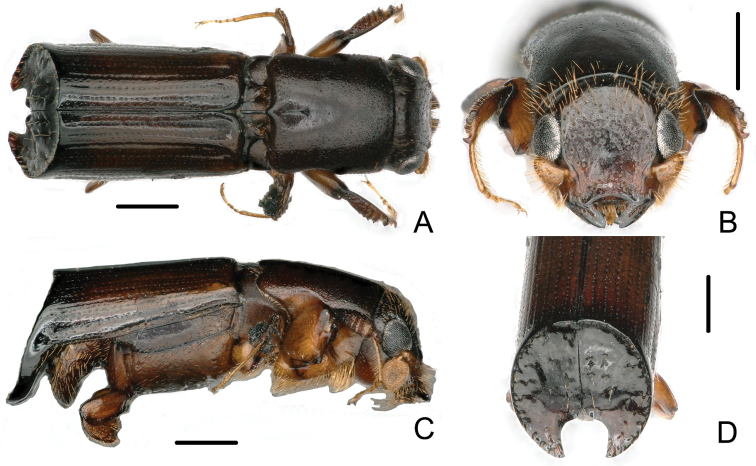
Male of *Crossotarsus beaveri* sp. nov. **A** dorsal view **B** head **C** lateral view **D** declivity. Scale bars: 0.5 mm.

**Figure 2.**
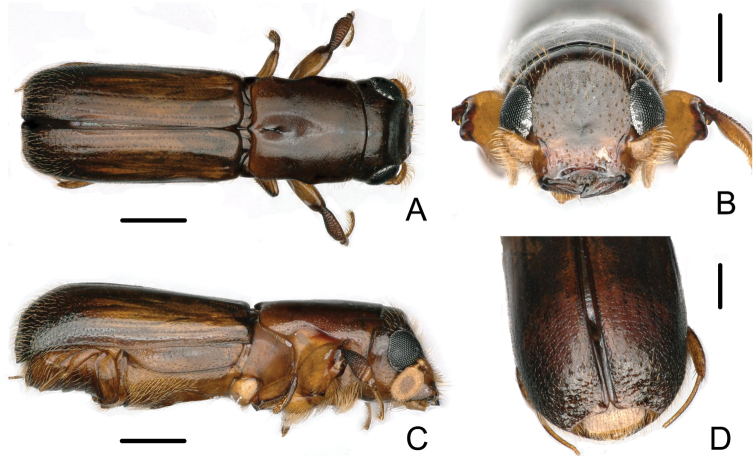
Female of *Crossotarsus beaveri* sp. nov. **A** dorsal view **B** head **C** lateral view **D** declivity. Scale bars: 0.5 mm.

#### Description.

**Male.** 3.58–4.01 mm long (mean = 3.78; *n* = 20); 2.75–2.95 times as long as wide. Head and pronotum dark brown, disc of elytra reddish brown becoming dark brown, declivity of elytra nearly black.

***Head.*** Frons flat, slightly shining, with irregular large punctures; finely, sparsely punctured above the epistoma, bearing bristly, erect, long setae, weakly concave, smooth around short median line, upper part of frons with scattered, coarse punctures, the punctures with moderate, semierect, dorsally directed setae. Antennal scape clavate with scattered, forwardly directed hairs on apical half; club oval, flattened, evenly covered with short setae. Labial palps two-segmented, with basal segments fused along the midline.

***Pronotum.*** About 1.2 times longer than wide, shining, no mycangial pores, the lateral femoral grooves angulate anteriorly, pronotum widest in front of the grooves, with finely, scattered, irregular punctures, a few semierect backwardly pointed hairs close to anterior margin, median line extending about 1/4 from base.

***Scutellum.*** Depressed below level of elytra, with a median longitudinal groove between lateral carinae.

***Elytra.*** About 2.0 times as long as wide, about 1.4 times as long as pronotum. Surface of disc smooth, shining, striae distinctly impressed for almost their entire length, except striae 6 and 7, other striae with circular, distinct, shallow punctures, the bases of striae 1 and 2, striae 3 and 4, respectively, conjoint, more impressed; interstriae slightly raised on disc, interstriae 1, 3 and 5 distinctly raised and conjoint at base, interstriae 8 and 9 fused at apex of disc, forming ventral, rounded angle; cylindrical declivity obliquely truncate, acutely margined all around except at sutural apex, strongly concave, forming a cup-like structure, surface shining, with 4 rows of longitudinal granules bearing erect, long, golden setae, a row of sparse, medially directed, erect golden setae at the inner margin of declivity, elytral apex broadly emarginate, the main emargination approximately U-shaped, about as wide as deep, extending about 1/3 of the height of the declivity, at its inner end a much smaller, V-shaped second emargination (Fig. 1A, D).

**Figure 3.**
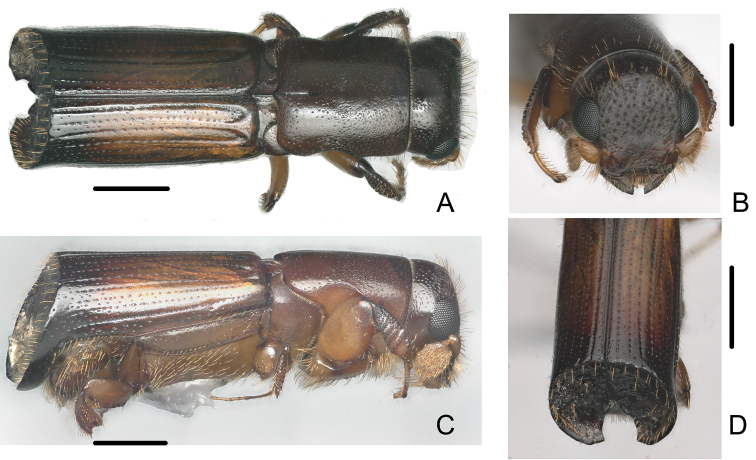
Male of *Crossotarsus brevis* (Browne) **A** dorsal view **B** head **C** lateral view **D** declivity. Scale bars: 0.5 mm.

***Protibia*.** Five transverse carinae at tibial apex, transverse rugae at base.

***Abdomen.*** Abdominal ventrites 1–4 moderately finely punctured, with irregular rows of erect, short hairs at both sides posteriorly, ventrite 5 strongly concave at middle, with dense, large, circular punctures.

**Female.** 3.64–4.42 mm long (mean = 3.96 mm; *n* = 20); 2.79–2.93 times as long as wide. Head and pronotum brown, disc of elytra reddish brown becoming dark brown to apex.

***Head.*** Similar to male, but frons flatter, very shining, smooth, with shallow, small punctures; finely, sparsely punctured above the epistoma, bearing bristly, erect, long setae; very shallowly concave in median line, upper part of frons with scattered, shallow, small punctures, the punctures with moderate, semierect, dorsally directed setae.

***Pronotum.*** Similar to male.

***Elytra.*** About 1.8 times as long as wide, about 1.5 times as long as pronotum, sides subparallel. Similar to male, but disc of elytra shining, with dense, longitudinal, semierect, backwardly pointed hairs at apex and declivity, striae weakly impressed, interstriae smoother, declivity vertical, a few irregularly granules, sparsely hairy.

***Protibia.*** Three transverse carinae at tibial apex, fine, confused granules at base.

***Abdomen.*** Surface of abdominal ventrites smooth, rounded, sparsely hairy, ventrite 5 without concavity, punctures shallow.

#### Etymology.

The species is named for Roger A. Beaver to honor his contributions to the study of platypodines and scolytines.

#### Host plants.

*Castanopsis carlesii* (Hemsl.) Hayata, *C. fargesii* Franch. (Fagaceae), *Liquidambar formosana* Hance (Altingiaceae), *Phoebe zhennan* S.K.Lee & F.N.Wei (Lauraceae), *Paulownia fortunei* (Seem.) Hemsl. (Paulowniaceae), *Vernicia montana* Lour. (Euphorbiaceae).

#### Distribution.

China (Jiangxi, Fujian).

#### Diagnosis.

The species is placed in *Crossotarsus* because it possesses a combination of characters similar to that cited in the introduction.

*Crossotarsus beaveri* is very similar to *Crossotarsus brevis* (Browne, 1975) (new combination, see below) and *Crossotarsus platypoides* (Browne, 1955). They can be easily distinguished from other *Crossotarsus* species by the male elytral apex truncate with a large, circular, concave declivity. The elytral apex of male of *C. beaveri* and that of *C. brevis* possess a deep, acutely margined declivity, with a broad, almost circular, apical emargination.

### Key to the species of *Crossotarsus* with a circular, truncate elytral declivity

**Table:** 

1	Male elytral apex truncate, with a circular, shallow, concave, bluntly margined declivity; sutural apex of declivity slightly dehiscent without apical emargination. Female smaller and stouter, 2.60–2.70 mm long, 2.70–2.75 times as long as wide	***C. platypoides* Browne**
–	Male elytral apex truncate, with a circular, deep, concave, acutely margined declivity, with a broad, almost circular, apical emargination. Female larger and more elongate, 3.00–3.90 mm long, 2.79–3.44 times as long as wide	**2**
2	Male striae weakly impressed on disc of elytra (Fig. 1A); declivity gradually, obliquely truncate, its face shining, cylindrical, apex rounded with a double sutural emargination, borders of inner emargination weakly elevated, outer emargination forming pointed angles; surface of declivity with 4 longitudinal rows of granules, bearing erect, long golden setae (Fig. 1D). Female frons flat, more shining, smoother, very shallowly concave in median line; dense, shallow, small punctures bearing semierect hairs on upper part; almost flat above the epistoma below median line (Fig. 2B); striae weakly impressed on disc of elytra (Fig. 2A). 3.64–3.90 mm long	***C. beaveri* sp. nov.**
–	Male striae moderately impressed on disc of elytra (Fig. 3A); declivity abruptly, vertically truncate, its face subnitid, cylindrical, apex rounded with a double sutural emargination, borders of inner emargination distinctly elevated and dilated, outer emargination forming obtuse angles; surface of declivity with sparse, obscure granules, bearing erect, long golden setae (Fig. 3D). Female frons slightly shining, reticulate, very distinctly concave, smooth around median line; dense, deep, large punctures bearing semierect hairs on upper part; weakly, irregularly impressed above the epistoma below median line (Fig. 4B); striae moderately impressed on disc of elytra (Fig. 4A). 2.96–3.44 mm long	***C. brevis* (Browne)**

### Crossotarsus brevis

Taxon classificationAnimaliaColeopteraCurculionidae

(Browne, 1975) comb. nov.

85E5F3D9-228A-5B77-B19F-A7B701DD74B2

Figures 3 , 4

Platypus brevis Browne: Beaver and Browne 1975: 306.
Dinoplatypus brevis Browne: Beaver 1998:184.

#### Material examined.

7 males, 5 females (JXAU); 1 male, 1 female (RAB): China: Yunnan Province, Xishuangbanna Dai Autonomous Prefecture, Jinghong City, Damanmi Village, 22°02'50"N, 100°48'27"E, ca 580 m, 20.I.2018, log dissection, host unknown, Shengchang Lai leg.

**Figure 4.**
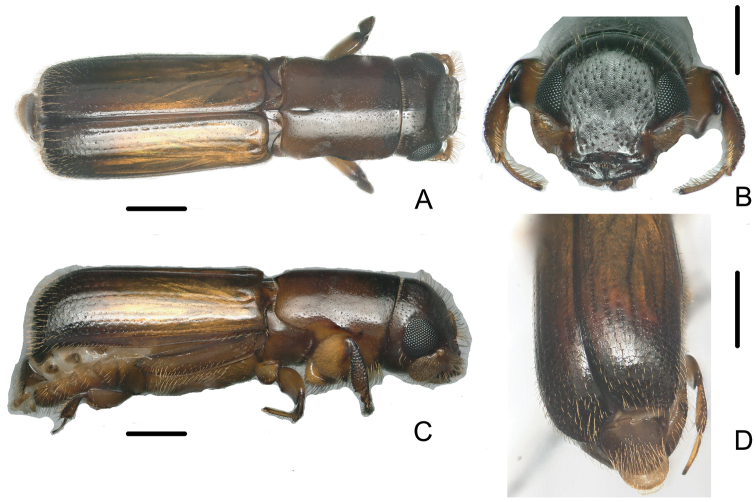
Female of *Crossotarsus brevis* (Browne) **A** dorsal view **B** head **C** lateral view **D** declivity. Scale bars: 0.5 mm.

#### Taxonomy.

The specimens in the RAB were identified by comparison to a paratype *C. brevis*, which is also in the RAB. Browne put this species in *Platypus* Herbst, noting that the apical emargination of the elytra was rather similar to that of *Platypus caliculus*Chapuis 1865 (Beaver and Browne 1975). In fact, *C. brevis* has the typical characters of *Crossotarsus*: labial palps two-segmented, with basal segments fused in the midline, whereas *Platypus* has the labial palps three-segmented, with separate basal segments. Beaver (1998) transferred the species from *Platypus* to *Dinoplatypus* Wood following Wood’s (1993) attempt to split the genus *Platypus*. Wood diagnosed *Dinoplatypus* largely on the basis of the circular, truncate, elytral declivity of the male, with the sutural apex emarginate. However, this is an adaptive character of the declivity which has evolved independently more than once in the Platypodinae, as it has in the Scolytinae (Hulcr et al. 2015). Molecular phylogenetic study also shows that the few morphological characters used by Wood (1993) to erect several groups of Neotropical and Indo-Malayan/Australasian species in Platypodini to new genera are not sufficiently diagnosable for all those groups (Jordal 2015).

Browne (1961) and Beaver and Sanguansub (2015) suggested that the adult generic characters of primary value in *Crossotarsus* included the structure of the labial and maxillary palps, the form of the pronotum, the sexual dimorphism of the protibia, and various modifications of the abdominal sternites in the male. Based on the two-segmented labial palps, the lateral pronotal emarginations angulate anteriorly, the pronotum without mycangial pores, and the sexual dimorphism of the protibiae, *Platypus brevis* belongs in the genus *Crossotarsus*, and is here transferred to that genus.

#### Distribution.

Thailand (Beaver and Liu 2013). New to China (Yunnan).

#### Host.

*Castanopsis* sp. (Fagaceae) (Beaver and Liu 2013).

### New record

#### Crossotarsus emorsus

Taxon classificationAnimaliaColeopteraCurculionidae

Beeson, 1937

799EF04F-6B6F-5A16-BC37-64B93E0C03F1

Figures 5 , 6

Crossotarsus emorsus Beeson, 1937: 87.

##### Material examined.

4 males, 1 female (JXAU) China: Yunnan Province, Xishuangbanna Dai Autonomous Prefecture, Jinghong City, Nabanhe River Watershed National Nature Reserve, Guomenshan, ca 1030 m, 22°14'46"N, 100°36'10"E, 27.I.2018, log dissection, host *Dalbergia assamica*, Shengchang Lai leg.; 1 male, 1 female (RAB); 1 male (JXAU) China: Yunnan Province, Xishuangbanna Dai Autonomous Prefecture, Jinghong City, Damanmi Village, ca 580 m, 22°02'50"N, 100°48'27"E, 20.I.2018, log dissection, host *Cassia siamea*, Shengchang Lai leg.

**Figure 5.**
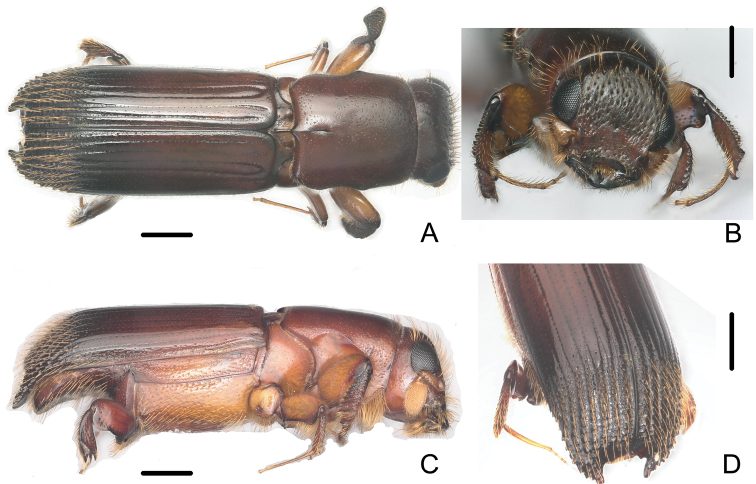
Male of *Crossotarsus emorsus* Beeson **A** dorsal view **B** head **C** lateral view **D** declivity. Scale bars: 0.5 mm.

##### Diagnosis.

*C. emorsus* is similar to *C. terminatus* but can be distinguished using the characters given in Table 3.

**Table 3.** Diagnostic characters separating *Crossotarsus emorsus* and *Crossotarsus terminatus*.

	*C. emorsus*	*C. terminatus*
Body size	Male size 4.56–4.80 mm long, 3.20–3.42 times as long as wide;	Male size 3.32–3.40 mm long, 2.78–3.00 times as long as wide;
	Female size 4.8–5.34 mm long, 3.38–3.43 times as long as wide.	Female size 3.44–3.58 mm long, 2.87–2.93 times as long as wide.
Frons	Male frons almost flat, with shallower, irregularly placed punctures; circularly concave in median line.	Male frons coarser, with deeper, irregularly placed punctures; linearly concave in median line.
	Female frons almost flat, without concave around median line.	Female frons concave forming a big, circular impression around concave median line.
Elytra	Male without lateral emargination at declivity base, semicircular lateral borders with serrated, lateral tubercles.	Male with lateral emargination at declivity base, semicircular lateral borders rounded, without distinct serrated, lateral tubercles.

##### Distribution.

Myanmar, Thailand, Laos (Beaver and Liu 2013; Beaver 2016). New to China (Yunnan).

##### Host.

The species is recorded from trees in the families Lecythidaceae, Fabaceae, Sterculiaceae and Verbenaceae (Beeson 1937), and is presumably polyphagous (Beaver 2016). Host plants recorded here are: *Senna siamea* (Lam.) H.S.Irwin & Barneby and *Dalbergia assamica* Benth. (Fabaceae).

**Figure 6.**
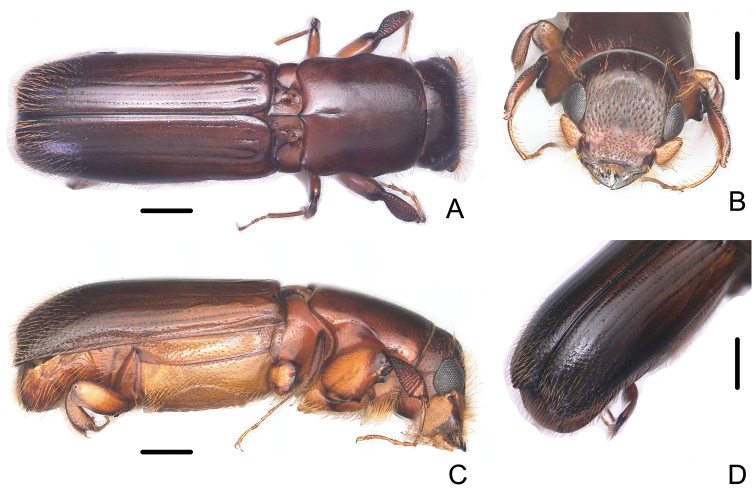
Female of *Crossotarsus emorsus* Beeson **A** dorsal view **B** head **C** lateral view **D** declivity. Scale bars: 0.5 mm.

##### Molecular data.

**Figure 7.**
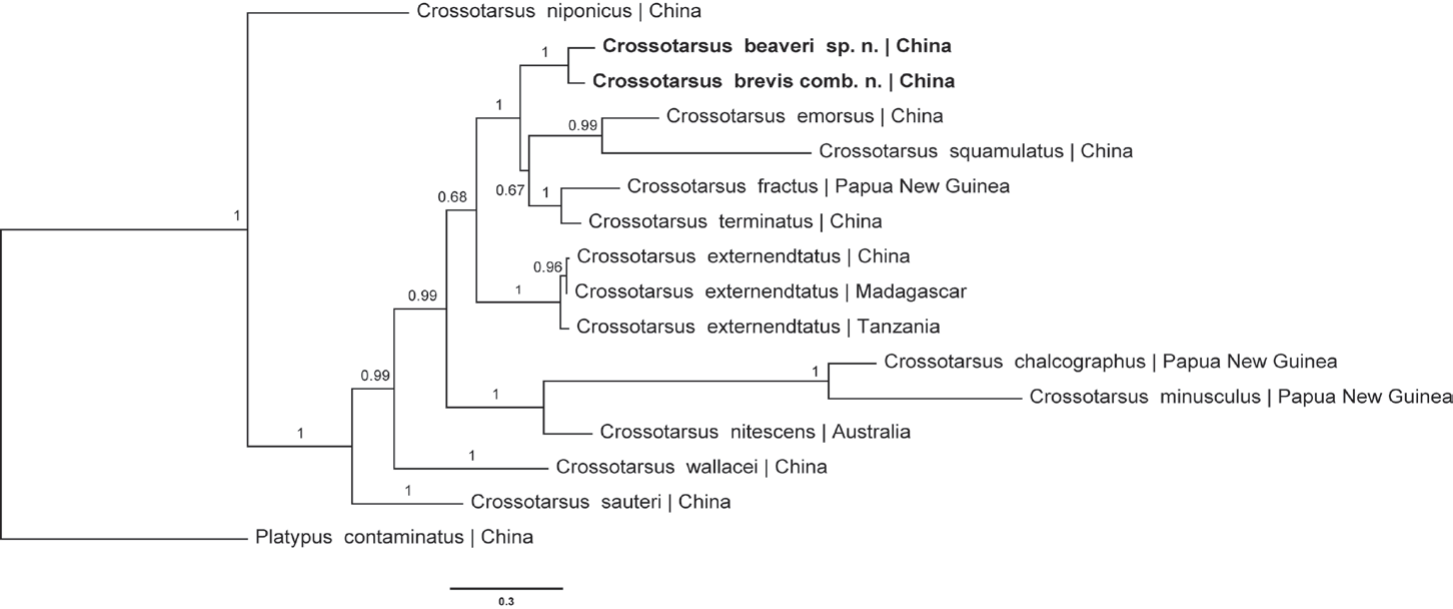
Tree topology resulting from Bayesian analysis of four genes. Posterior probabilities are given on the nodes. New species and new combination in boldface.

The phylogenetic tree for analyzing the evolutionary relationships of 13 taxa including the ingroups (*Crossotarsus* species) and the outgroups (*P. contaminatus*) was constructed based on four genes (Fig. 7). The BI tree shows the new species (*C. beaveri*) and the new combination (*C. brevis*) forming a clade, with high node support. These group with Schedl’s (1972a) ‘*Crossotarsi coleoptrati*’ (*C. fractus* Sampson, 1912, *C. squamulatus* and *C. terminatus*) and cluster with all remaining *Crossotarsus* species. It confirms that the taxonomic changes and the relationship of *C. brevis* and *C. brevis* are correct. It also indicates that *C. emorsus*, *C. fractus*, *C. squamulatus* and *C. terminatus* should be considered distinct species (as by Beaver and Liu 2013), and not considered synonyms or subspecies (Schedl 1972a).

## Discussion

*Crossotarsus beaveri* is clearly related to *C. brevis*. They are the sister lineage to the *Crossotarsi coleoptrati* group, not the genus *Dinoplatypus*. This is a good example that declivity in males usually is an adaptive character and not of generic significance. We consider that the morphologically diagnosable characters of the genus *Crossotarsus* should refer to the summary by Browne (1961) and Beaver and Sanguansub (2015, 2020), as introduction.

The genus *Crossotarsus* is one of the largest genera of Platypodinae, with more than 100 species. Although there are 13 previously recorded species of Chinese *Crossotarsus* (Yin and Huang 1987; Yin et al. 2002; Zhang et al. 2008), many additional species have been reported from countries neighboring China (Beaver and Shih 2003; Goto 2009; Beaver and Liu 2013; Beaver 2016) which still have not been found in China. This strongly indicates that many more species remain to be discovered, especially on the Chinese mainland. *Crossotarsus* is monophyletic in the latest molecular phylogeny (Jordal 2015). There are few molecular data for the genus in GenBank, less than 10% of the whole. More taxonomic samples are needed.

## Supplementary Material

XML Treatment for Crossotarsus beaveri

XML Treatment for Crossotarsus brevis

XML Treatment for Crossotarsus emorsus
